# Post-Transplant Lymphoproliferative Disorder: A Rare Case of CNS Involvement following Renal Transplant

**DOI:** 10.3390/jcm11237058

**Published:** 2022-11-29

**Authors:** Austin Feindt, Montserrat Lara-Velazquez, Ahmad Alkhasawneh, Dinesh Rao, Raafat Makary, Keith Dombrowski, Daryoush Tavanaiepour, Gazanfar Rahmathulla

**Affiliations:** 1 School of Medicine, University of South Florida School of Medicine, 12901 Bruce B Downs Blvd., Tampa, FL 33612, USA; 2 Department of Neurological Surgery, University of Florida College of Medicine, 653-1 8th St. W, Jacksonville, FL 32209, USA; 3 Department of Pathology, University of Florida College of Medicine, 8th St. W, Jacksonville, FL 32209, USA; 4 Department of Radiology, University of Florida College of Medicine, 8th St. W, Jacksonville, FL 32209, USA; 5 Department of Neurosurgery and Brain Repair, USF Health, 2 Tampa General Cir, Tampa, FL 33606, USA

**Keywords:** lymphoproliferative disorder, organ transplantation, Epstein–Barr virus infections

## Abstract

Background: Post-transplant lymphoproliferative disorders (PTLD) are rare immunosuppression complications affecting 5% of transplant patients. Isolated central nervous system (CNS)-PTLD without nodal or extra-nodal organ involvement is rarely reported and is difficult to diagnose due to the non-specific clinical manifestations and imaging features overlapping with other common CNS lesions. Case presentation: We present a case of a 72-year-old female subjected to a renal transplant 11 years ago with progressively worsening headaches and confusion. Imaging revealed vasogenic edema in the left frontal and bilateral temporal lobes. She was subjected to a craniotomy and excisional biopsy to obtain tissue for diagnostic and therapeutic interventions. Pathology examination showed atypical EBV-positive lymphoplasmacytic infiltrate, consistent with Polymorphic type PTLD. Conclusions: Patients diagnosed with PTLD need to have close monitoring of immunosuppressive medications while in the hospital. Early diagnosis is essential for patient survival with PTLD, as their health can deteriorate fast.

## 1. Introduction

Post-transplant lymphoproliferative disorders (PTLD) are a rare complication of immunosuppression that affects approximately 5% of transplanted patients. PTLD was first described in renal transplant recipients more than 50 years ago.

Solid organ transplantation recipients have a fivefold increased risk of lymphoproliferative disorders compared with the general population. As the consequence of immunosuppression, the Epstein–Barr virus (EBV) infection of lymphocytes (usually from the host; subclinical or infectious mononucleosis) immortalizes B cells [1,2]. The extended life of EBV-infected B cells increases the risk of acquiring molecular aberrations that confer growth advantage. Meanwhile, the immunosuppressed patients are unable to mount effective T cell cytotoxicity, which can cause uncontrolled polyclonal lymphoid proliferation [2,3].

Monoclonal populations may emerge and, with mutations, cause malignancy. The majority of mature lymphomas that occur are diffuse large B-cell lymphomas, but also rare subtypes like T-cell, Burkitt, plasmacytoid, and Hodgkin-like PTLD may arise [1,2]. Tumors of donor origin may be more indolent than those of recipient origin [1].

The incidence of PTLD varies among different transplant centers according to different immunosuppressive protocols, transplant types, and patient population demographics. The lowest incidence of PTLD is reported in renal and liver transplants (1–5%) and the highest incidence in intestinal, lung, and multi-organ transplants (5–20%) [3]. High incidence has been related to the greater immunosuppression necessary to protect the transplant in the latter group [3,4,5,6].

PTLD encompasses a spectrum ranging from EBV-driven infectious mononucleosis-type polyclonal proliferations to EBV positive or negative proliferations indistinguishable from a subset of B cell or less often T cell lymphomas similar to their counterpart in immunocompetent individuals.

Organs involved by PTLD in decreasing frequency are lymph nodes, GI tract, lungs, liver (common), bone marrow (uncommon), and rarely the central nervous system (CNS) or peripheral blood. Bone marrow recipients tend to have a widespread disease [5,6,7]. 

Isolated CNS-PTLD without nodal or extra-nodal organ involvement is rarely reported and is difficult to diagnose due to the non-specific clinical manifestations and imaging features overlapping with other common CNS lesions [6]. CNS-PTLD lesions are usually multifocal, with supratentorial and lobar involvement. Due to its not-so-common clinical and radiological presentation, CNS-PTLD is often not considered as first diagnosis by physicians not familiar with the pathology. Here, we present such a case with a fatal outcome and provide the key points that will help to reduce the differential diagnosis list to provide timely and targeted treatment.

## 2. Case Presentation

A 72-year-old female with a past medical history of a renal transplant 11 years ago was admitted to our hospital with progressively worsening headaches and confusion. She had unremarkable medical and surgical history otherwise. She was induced with dacrolimus and methylprednisolone and had been on maintenance immunosuppressive therapy with tacrolimus 1.5 mg in the morning and 1 mg at night, mycophenolate 500 mg twice a day, and prednisone 7.5 mg once a day, with regular follow-ups.

Screening for EBV was not performed when she was transplanted. Furthermore, at that time, she had a negative BK-PCR and a positive result for cytomegalovirus (CMV). The donor of the kidney was CMV-negative.

Her clinical examination did not show any focal motor or sensory deficits, and she had intact reflexes and a normal gait. Her mini-mental state examination revealed confusion and intermittent disorientation to person and place. 

**Figure 1:**
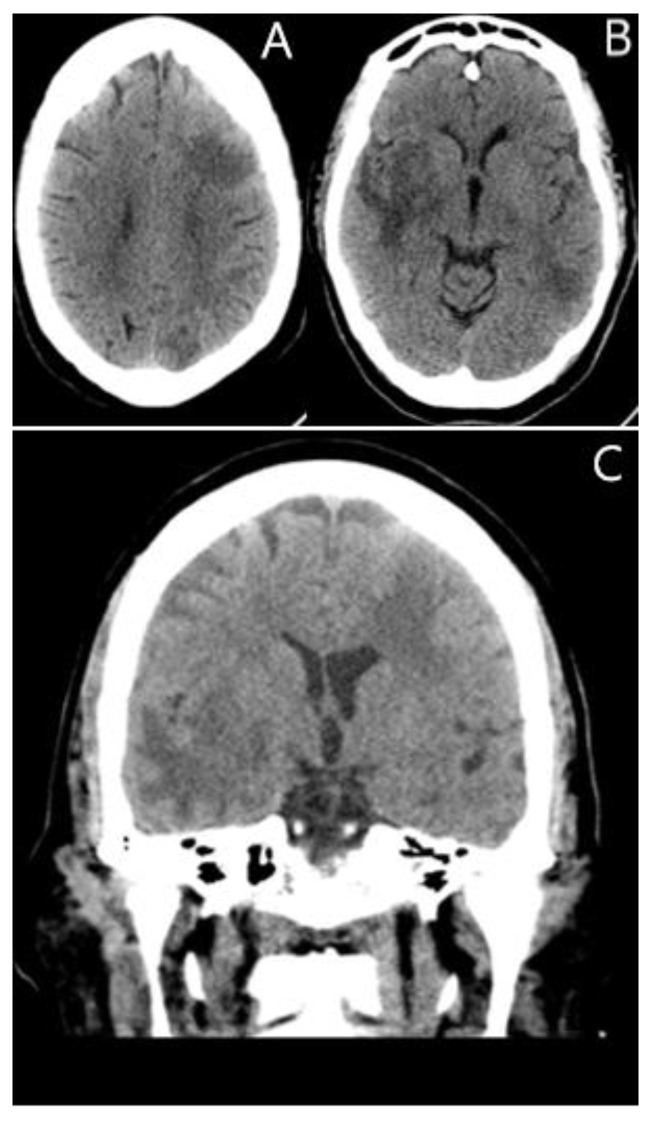
(**A–C**) Unenhanced Axial and Coronal CT images demonstrating vasogenic edema with a mass effect within the left frontal and bilateral temporal lobes. There is mild compression of the left lateral ventricle secondary to a left frontal lesion (**C**).

Further imaging with computed tomography (CT) revealed vasogenic edema in the left frontal and bilateral temporal lobes (Figure 1).

**Figure 2:**
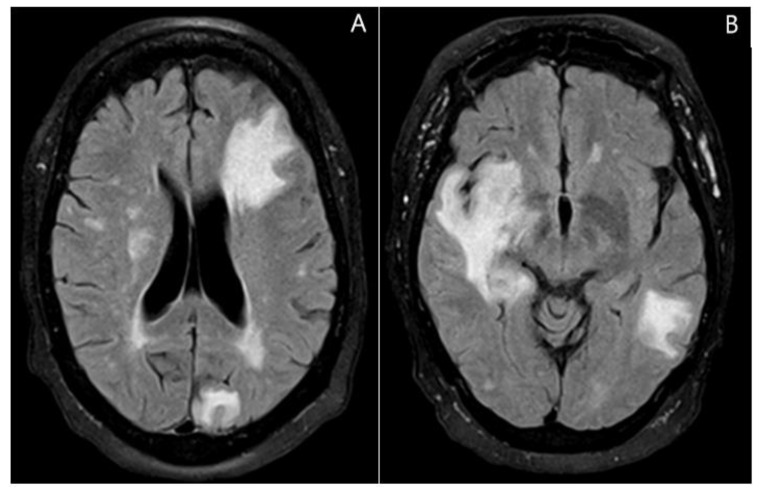
(**A**) Axial MRI FLAIR image demonstrating a hyperintense signal in the left frontal region left occipital and changes in a patchy manner occurring bilaterally. (**B**) There is a vasogenic edema and mass effect within the anterior right temporal lobe and a posterior left temporal lobe involving the left temporal stem and mesial temporal lobe.

**Figure 3:**
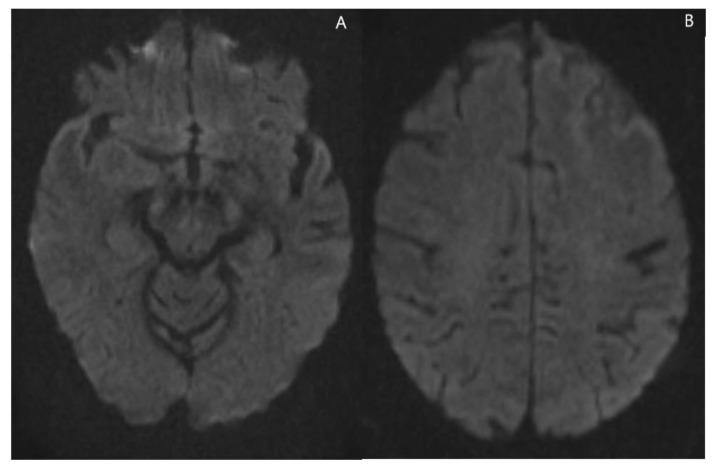
(**A**,**B**) Axial diffusion-weighted MRI demonstrating no restricted diffusion of the dominant right temporal and left frontal lesions. The other lesions (not shown) demonstrated no restricted diffusion.

Magnetic Resonance Imaging (MRI) with contrast revealed multiple lesions present in the left frontal, left parietal, and anterior left temporal lobes with the frontal lesions having significant vasogenic edema and mass effect (Figure 2 and Figure 3). 

**Figure 4:**
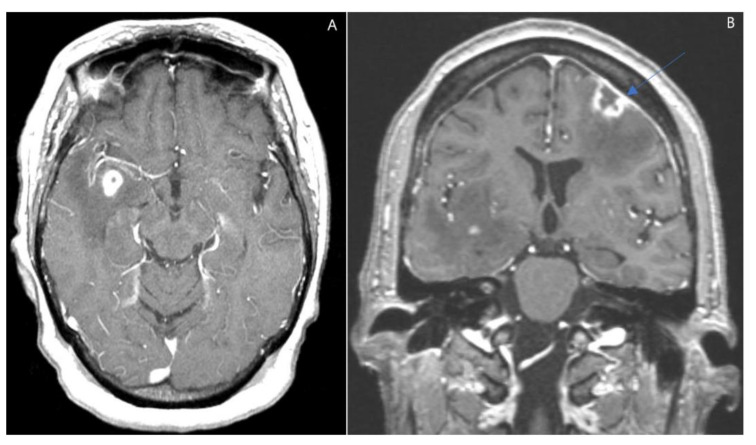
(**A,B**) Axial and Coronal enhanced T1 weighted MRI. There are peripherally enhancing lesions within the left frontal and right temporal lobes and pachymeningeal enhancement (blue arrow, **B**) overlying the left frontal lesion.

**Figure 5:**
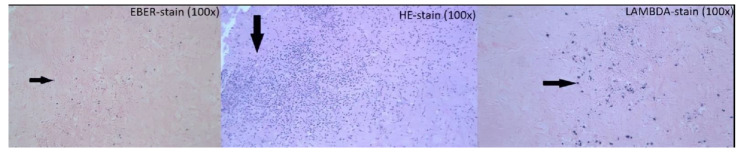
PTLD of the brain. Brain tissue with atypical lymphoplasmacytic infiltrate (arrow), H&E stain 100×. The plasma cells are lambda restricted (arrow), consistent with lymphoproliferative disorder (in situ hybridization stain for lambda, 100×). In situ hybridization for EBER shows scattered positive lymphocytes (arrow), which supports the diagnosis of post-transplant lymphoproliferative disorder PTLD (100×).

Following her imaging, there were no accessible lesions from which a biopsy could be obtained. We decided to perform a craniotomy and excision biopsy to obtain tissue for diagnostic and therapeutic interventions, as the differential included intracranial metastatic lesions as well as intracranial abscess. The left frontal craniotomy was performed directly over the frontal dural lesion with excision and central biopsy (Figure 4). Pathology examination showed atypical EBV-positive lymphoplasmacytic infiltrate, consistent with polymorphic type PTLD (Figure 5). Bone Marrow biopsy was negative for lymphoma. Her EBV antibody profile was positive for IgG and negative for IgM, with a negative EBV-PCR. HSV-PCR was positive. Her lactate dehydrogenase levels were between 280 to 576 U/L while hospitalized. Spectroscopy was pendent.

The patient was started on rituximab (RTX) along with reduction in her immunosuppressive medication (she was kept on tacrolimus 0.5 mg two times a day and dexamethasone 4 mg oral every 8 h). A follow up head CT showed no change in the multiple intraparenchymal lesions. Further work up included a CT of the chest, abdomen, and pelvis, which showed no other sites of disease. MRI on the spine showed complete severe degenerative disease, with no lymphoma involvement of the spinal cord noted in the study. In addition to the RTX, she required institution of proton beam therapy for her intracranial lesions. Unfortunately, she had poor clinical response to therapy and experienced multiple medical complications (sepsis, thrush, HSV gingivostomatitis, and perianal HSV). Antibiotics were started for sepsis; however, she did not respond to treatment, declined quickly, and unfortunately died. Because of this, we were not able to perform additional studies and obtain an informed consent. However, we have made sure that all the presented information has been de-identified.

## 3. Discussion

The clinical manifestation of PTLD may appear as early as 6 weeks after transplantation, but most cases manifest after years. The neurological signs and symptoms in CNS-PTLD patients are variable and non-specific. In our case, the patient presented with progressively worsening headaches and altered mental status/confusion.

Neuroimaging modalities demonstrate the extent of neuroanatomical areas involved. MRI is superior to CT for the detection of disease. Lesions tend to be multifocal, lobar, and supratentorial, and rarely present in the infratentorial region or in the spinal cord. The main affected CNS structures include the lobes (88%), basal ganglia (39%), and the periventricular area (72%), followed by the brainstem and cerebellum [8,9,10]. Less commonly, there is the involvement of the corpus callosum, thalami, or concurrent meningeal/ependymal enhancement [9]. CNS-PTLD lesions typically enhance with gadolinium and appear with partial or diffuse rings (from central necrosis) to ill-defined enhancing margins with homogeneous or heterogeneous (more common) enhancement [9,10]. The MRI with contrast, in this case, revealed multiple lesions in the left frontal, left parietal, and anterior left temporal lobes with significant vasogenic edema and mass effect from the frontal lesions [10]. The imaging findings reflect features of hypercellular tumors prone to hemorrhage, cystic-necrotic changes, and surrounding edema. The imaging features of CNS-PTLD are similar to other more common CNS lesions including glioblastoma (GBM), metastatic disease, primary CNS lymphoma, abscess/ infections, with tumefactive demyelination, stroke, or neurosarcoidosis less likely [10,11,12,13]. However, in the clinical context of transplant immunosuppression, subtle differences in neuroimaging will raise suspicion about the rare possibility of CNS-PTLD among the differential diagnosis.

Key findings to help clinicians to identify PTLD with CNS involvements are described in Table 1. 

As an educational resource, we include the main features of pathologies that are part of the differential diagnosis of CNS-PTLD in Table 2.

PTLDs are sub-classified into four categories, based on morphologic, immunophenotypic, and molecular criteria, as early hyperplastic lesions, polymorphic, monomorphic, and classic Hodgkin lymphoma types [2,14]. Early-PTLD types may be plasmacytic or florid follicular or infectious mononucleosis-like lymphoid hyperplasia. The polymorphic type is histologically characterized by a full spectrum of lymphoid maturation including immunoblasts, plasma cells, and small to medium B cell and T cell lymphocytes, and is often EBV (+) [15]. Among the histologic subtype of PTLD, the polymorphic PTLD accounts for about 19% [16]. The monomorphic PTLD subtype is composed by B, T, or NK-cell type neoplasms. The B cell lymphomas group includes the diffuse large B cell lymphoma, the Burkitt lymphoma, the plasma cell myeloma, or the plasmacytoma-like lesion. The T-cell lymphoma group includes the peripheral T cell lymphoma-NOS, the hepatosplenic T cell lymphoma, and others [16,17,18]. The majority of PTLD cases are B cell and the least are T/NK cell or Hodgkin lymphoma (5–10%) [18]. CNS-PTLD are usually of the monomorphic type, but some cases are polymorphic. Monomorphic CNS-PTLD resembles diffuse large B-cell lymphomas with a tendency for perivascular growth and is more commonly of non-germinal center cell origin (activated B cell-like), a subtype that has a worse prognosis. PTLD of T cell lineage has rarely been reported in the CNS [2]. The histologic examination of the frontal lesions in this case was consistent with the polymorphic type. The bone marrow biopsy was negative for lymphoma or other lymphoproliferative disorders. 

The prognosis and overall survival are significantly affected by several risk factors in univariate analysis, including patient gender, age at transplant and PTLD diagnosis, multiple acute rejections prior to PTLD diagnosis, IPI score (international prognostic index), allograft type, recipient EBV status, PTLD sub-type, extra-nodal site, immunosuppressive drug regimen at diagnosis, and initial treatment best response. Recently, tumor microenvironment (TME) and tumor infiltrating lymphocytes (TIL) were also described as important prognostic factors in patients with monomorphic PTLD. Low TIL count was described to be associated with progression free survival and overall survival at 2 years [2]. Moreover, monomorphic PTLD patients can be stratified by PFS and OS in low, intermediate, and high risk with a scoring system with factors like TILs, age, LDH, stage, and CNS involvement [2].

The median time for diagnosis of primary CNS PTLD following transplantation is 4.4 years [18], and our patient developed late onset (more than 1-year post-transplant) polymorphic PTLD after 11 years. Other cases with late onset (after 10 years) post-transplantation have been documented by Cavaliere and Evens [4,5,6,7,8,9,10,11,12,13,14,15,16,17,18].

Epstein–Barr virus (EBV) is positive in about 70% of PTLD, particularly early PTLD, from the chronic immunosuppression of T cells immune function that eventually enhances EBV proliferation. The pathogenesis in EBV-negative PTLD is not clearly understood. Univariate analysis of 141 cases studied by Bisnoi et al. [19] showed that bone marrow involvement had a poor prognosis. No other extra nodal site including CNS was found to have a statistically significant hazard ratio. CMV status of recipient or donor and tumor EBER status were not significant [19]. The survival in CNS-PTLD is very variable ranging from months to years [3,4,5,6,7,8,9]. Multivariate analysis found age to be predictive of survival [8].

The first line in PTLD treatment is a prudent decrease or withdrawal of immunosuppressive therapy to avoid rejection of the transplanted organ [3,6,7,8,9,20]. Corticosteroids are used routinely as a substitute for immunosuppression. Polymorphic and less often monomorphic PTLD may also regress with a high risk of rejection leading to graft loss and death [17,20].

Treatment with additional therapies (chemotherapy or radiotherapy +/− anti-CD20) could be considered if the first line of treatment fails. However, the results of these therapies have been heterogeneous. Methotrexate is more commonly used intravenously or less commonly intrathecally [3,8,9]. Rituximab and/or cytarabine with cranial radiation therapy results have been promising, with a trend for improved progression-free survival [10]. Treatment with antiviral therapies have shown mixed results. Novel treatment strategies for PTLD have emerged, including adoptive immunotherapy and therapeutics that target downstream signaling pathways of virus-encoded latent membrane protein-2A [11].

Mortality is generally higher in non-responders to immunosuppressive therapy modification and failures of treatment with first modality. In the case we presented, the patient was treated with chemotherapy, RTX, along with a reduction in immunosuppressive medication and proton beam therapy for her intracranial lesions. Unfortunately, despite the aggressive treatment, the clinical response was poor with systemic decline.

In a series of 34 primary CNS PTLD, 23% developed PTLD ≥10 years after transplant [18]. One of the risk factors for CND PTLD in our patient is age >60. In addition, the donor for the patients’ renal transplants had not been screened for the presence of EBV prior to transplantation. Although this diagnosis can be difficult for lymph node biopsy, our patient’s biopsy showed a mixture of infiltrating lymphoid and plasma cells that formed destructive CNS lesions, and do not fulfill the criteria for monomorphic PTLD. In addition, staining with EBER helped in establishing the diagnosis, which is found in about 2/3 of PTLD cases. 

## 4. Conclusions

CNS-PTLD is a rare entity with clinical and radiological characteristics that can resemble other pathologies. Thus, it can be challenging for physicians unfamiliar with the disease. Due to its special location, early diagnosis of CNS-PTLD is essential for patient prognosis. More studies need to be performed on the plan of care and treatment to accurately diagnose and treat PTLD that is EBV positive versus PTLD that is EBV negative. Although the current standard treatment is formed by two different aggressive approaches (Chemotherapy with Rituximab), this combination is not successful to eradicate PTLD with CNS involvement. The mortality rates of Early and Late-Onset PTLD remain very high, and thus it is very important to learn more about the disorder and to find short- and long-term successful treatments for PTLD.

## Figures and Tables

**Table 1:** Clinical, imaging, and spectroscopy findings of CNS-PTLD [8,10,17,18].

Clinical Findings	Imaging Findings	Spectroscopy Findings
Post-transplanted patients of solid organs, stem cells or bone marrow	Partial or diffuse ring enhancement pattern	Increased levels of choline, lipid, and lactate dehydrogenase
It can be present as soon as 6 weeks or >10 years post transplantation	Irregular/ill-defined heterogenous margin of enhancement	Preserve or decrease levels of N-acetylaspartate (NAA) Elevated choline (Cho)/creatinine (Cr) ratio Decreased NAA/Cho ratio
Usually, multifocal lesions	Hypercellular tumors with a mixed pattern of cystic- central necrosis and hemorrhagic areas	
Lobes, basal ganglia, periventricular, brainstem, and cerebellum involvement	Lower perfusion than lymphomas	
	Some lesions surrounded by edema	
	Apparent Diffusion Coefficient (ADC) elevated with areas of restricted diffusion. Susceptibility weighted imaging shows a peripheral pattern of punctate hypo intensities	

**Table 2:** Differential features of pathologies that resemble CNS-PTLD.

AIDS Lymphomas	PCNS Lymphomas	Glioblastoma	Brain Abscess	Metastasis
Multifocal lesions located in the basal ganglia and corpus callosum that show an heterogenous ring enhancement pattern with irregular margins. These lesions show an ADC that is slightly elevated, with increased Cho and decreased NAA and Cr in spectroscopy.	Unifocal periventricular lesions that usually invade the corpus callosum. These lesions show a diffuse and homogeneous solid enhancement pattern and defined margins. PCNS lesions have a lower and homogenous ADC, show elevated perfusion rates, with elevated Cho, lipid and LDH, and decreased NAA and Cr in spectroscopy.	An enhancing lesion with high perfusion that usually invades the corpus callosum; it also tends to show central necrosis and hemorrhagic areas.	Usually, a lesion with a thin rim of enhancement, with high diffusion and restricted ADC in the central area. In the periphery, an abscess shows a low diffusion signal with elevated ADC. The spectroscopy shows amino acids like valine, leucine, isoleucine, acetate, alanine, and succinate present in the abscess cavity	Are enhancing lesions with high perfusion rates. Location of the masses depends on the primary tumor, but in the CNS, they usually do not invade basal ganglia, periventricular areas, or the thalamic regions.
